# Unraveling Spatiotemporal Heterogeneity and Driving Mechanism of Vegetation Recovery in Co‐Seismic Landslide Areas via Nonlinear Trajectories and Bayesian Networks

**DOI:** 10.1002/ece3.74117

**Published:** 2026-07-29

**Authors:** Mingxuan Wan, Wei Zhao, Jiujiang Wu, Yanqing Yang, Junli Zhao

**Affiliations:** ^1^ Institute of Mountain Hazards and Environment Chinese Academy of Sciences Chengdu China; ^2^ University of Chinese Academy of Sciences Beijing China

**Keywords:** Bayesian network, generalized additive model, Landsat, landslides, vegetation recovery

## Abstract

Earthquakes often trigger extensive landslides that strip large areas of vegetation, leaving barren surfaces and posing significant challenges to ecological restoration. A notable example is the 2008 Wenchuan earthquake, which induced numerous co‐seismic landslides and created widespread bare areas that have hindered vegetation recovery. Understanding post‐disruption vegetation recovery patterns and their climatic drivers is therefore essential for targeted restoration efforts. This study establishes an integrated framework combining nonlinear trajectory analysis with Bayesian probabilistic inference to quantify vegetation recovery dynamics and identify key climatic controls. Using Landsat time‐series data (2008–2023), we applied a Generalized Additive Model (GAM)‐based nonlinear trajectory approach to extract recovery trajectories, identify trajectory‐derived recovery inflection points marking the transition from accelerated to decelerated recovery, and quantify spatial variations in recovery rates. A Bayesian Network (BN) was then used to quantify the influence of ecological memory and environmental drivers on recovery regimes, revealing critical temperature–moisture–radiation configurations and enabling scenario‐based evaluation of climatic constraints on regional recovery potential. GAM results indicate that most areas reached a critical recovery inflection point within 5–7 years, while delayed recovery was mainly observed in high‐elevation, rugged terrain. The BN further identified that a synergistic “warm–moist–moderate light” regime is necessary for fast recovery. Scenario simulations highlighted clear spatial differences in limiting factors: solar radiation is the dominant constraint in rugged highlands, whereas temperature and precipitation exert stronger controls in low‐elevation or moderately disturbed areas. This spatial variability suggests that restoration interventions should be tailored to local conditions to maximize efficiency. Overall, this study provides a transferable framework for diagnosing environmental constraints and guiding restoration strategies to enhance ecosystem recovery capacity in disaster‐prone mountainous landscapes.

## Graphical abstract

We developed an integrated GAM–Bayesian framework to diagnose the nonlinear recovery dynamics of vegetation in landslide‐affected areas. Our model reveals that most sites reached a key recovery inflection point within 5–7 years, with faster recovery occurring under warm, moist, and moderate light conditions. Scenario simulations further highlight that limiting factors vary spatially, emphasizing that restoration actions must be tailored to local conditions to maximize efficiency.

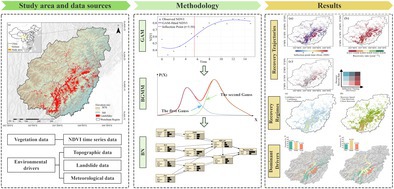

## 1 Introduction

Vegetation plays a fundamental role in maintaining ecosystem stability and mitigating geohazards in mountainous regions (Körner 2004; Shen et al. 2017; Zhou et al. 2008). Its root systems reinforce soil structure, reduce erosion, and enhance slope stability, forming a vital natural barrier against disasters (Gyssels et al. 2005; Morgan 2009). However, massive disturbances such as earthquakes and their secondary hazards can trigger catastrophic landscape transformations, severely altering ecosystem structure, function, and resilience (Turner 2010; Keefer 1984). The 2008 Wenchuan earthquake, for instance, induced tens of thousands of co‐seismic landslides, creating vast barren areas and leading to severe soil erosion, biodiversity loss, and disruption of key ecosystem services (Yang et al. 2018a; Dai et al. 2011; Wan et al. 2026). Such large‐scale disturbances not only weaken the natural defense against hazards but also disrupt the fundamental conditions required for vegetation recovery, posing long‐term challenges to ecosystem stability (Cui et al. 2011; Guariguata 1990; Xu et al. 2024).

In these severely disturbed environments, vegetation recovery typically follows complex, nonlinear trajectories rather than simple monotonic growth (Chen et al. 2022). From a dynamical‐systems perspective, strong disturbances such as co‐seismic landslides can push ecosystems into a low‐resilience state. Ecological resilience theory posits that the rate of recovery is a fundamental diagnostic of a system's capacity to buffer disturbances and rebuild its baseline state (Holling 1996). Based on this premise, we argue that the temporal dynamics of recovery rates offer a vital window into how recovery potential evolves post‐disturbance. Therefore, accurately characterizing temporal variations in recovery rates provides valuable insights into the dynamics of ecosystem re‐establishment and the progression of post‐disturbance recovery.

Currently, substantial researches have been conducted with satellite‐derived vegetation indices to quantify post‐disturbance vegetation recovery (Priya and Vani 2024; Yan et al. 2025). The foundational understanding of the spatial variability of the recovery process was mainly acquired from linear trend analyses or parametric growth models (Gan et al. 2019; Sun et al. 2021; Wang, Wang, et al. 2022; Yang et al. 2018b). However, such monotonic or time‐averaged approaches oversimplify the intrinsic nonlinear dynamics of recovery, particularly the transitional phases where recovery rates change—a key indicator of shifts in ecosystem recovery potential (Scheffer et al. 2001; Smith et al. 2022). In details, time‐series decomposition algorithms such as Breaks For Additive Season and Trend (BFAST) and Bayesian Estimator of Abrupt change, Seasonality, and Trend (BEAST) have been widely used to detect abrupt breakpoints associated with external disturbances (Verbesselt et al. 2010; Zhao et al. 2019). Nevertheless, these methods are primarily designed for change‐point detection rather than capturing the intrinsic curvature and temporal variability of a continuous recovery trajectory. In gradual post‐disturbance succession, the key scientific challenge shifts from identifying externally driven breakpoints to resolving the shape and curvature of the recovery trajectory, including changes in recovery rates over time. Consequently, a critical methodological gap remains in objectively delineating ecologically meaningful recovery stages from this continuous trajectory, which is essential for comparing driver impacts across heterogeneous recovery patterns and rates.

Furthermore, behind the recovery trajectory investigation, climatic drivers are frequently acknowledged as primary drivers of vegetation dynamics (Seidl et al. 2017; Shang et al. 2022; Su et al. 2025; Li, Gu, and Cao 2025). However, quantitatively disentangling their effects on post‐disturbance vegetation recovery remains challenging. Recent advances have leveraged multi‐year satellite observations and data‐driven models to assess the relative contributions of temperature, precipitation, and solar radiation to vegetation regrowth (Chen et al. 2024; Copeland et al. 2019; Yang et al. 2022). These studies consistently reveal that climatic influences are highly nonlinear and context‐dependent (Li, Zhao, et al. 2025; Zhao et al. 2026), interacting with topography, soil properties, and disturbance intensity. However, conventional attribution approaches—such as correlation or regression analyses—struggle to capture such nonlinear feedbacks and threshold responses, and are further constrained by their limited capacity to handle uncertainty in complex ecological systems (Zuur et al. 2010). Addressing these challenges requires probabilistic frameworks capable of representing conditional dependencies and uncertainty. The Bayesian Network (BN) provides such capability, enabling the modeling of multidimensional probabilistic relationships and facilitating scenario‐based inference (Aguilera et al. 2011; Uusitalo 2007). Although BN has been increasingly applied to ecological modeling and environmental decision‐making (Das and Chanda 2023; Hu et al. 2024; Jiang et al. 2023; Marcot et al. 2001), its use in diagnosing the climatic drivers that govern the emergence of distinct recovery regimes remains limited.

To address these gaps, this study aims to develop an integrated framework to characterize the nonlinear vegetation recovery following co‐seismic landslides and to identify the climatic controls driving the spatial divergence of recovery regimes. Taking the Wenchuan earthquake region as the study area, we first extracted post‐disturbance recovery trajectories using Landsat time series (2008–2023), detected ecological transition points marking the onset of accelerated regrowth, and quantified recovery rates to reveal their spatial differentiation. Based on the derived recovery regimes, we constructed a BN to link recovery regimes with multidimensional environmental drivers. This probabilistic approach enabled diagnostic assessments of the key hydrothermal–radiative configurations shaping each recovery regime and scenario‐based evaluations of how climatic constraints regulate regional recovery potentials. By integrating process‐oriented recovery characterization with probabilistic causal inference, this study provides new insights into the environmental controls and constraints on vegetation recovery and offers a scientific foundation for designing targeted restoration and climate‐adaptation strategies in disaster‐prone mountain regions.

## 2 Study Area and Data

### 2.1 Study Area

**FIGURE 1:**
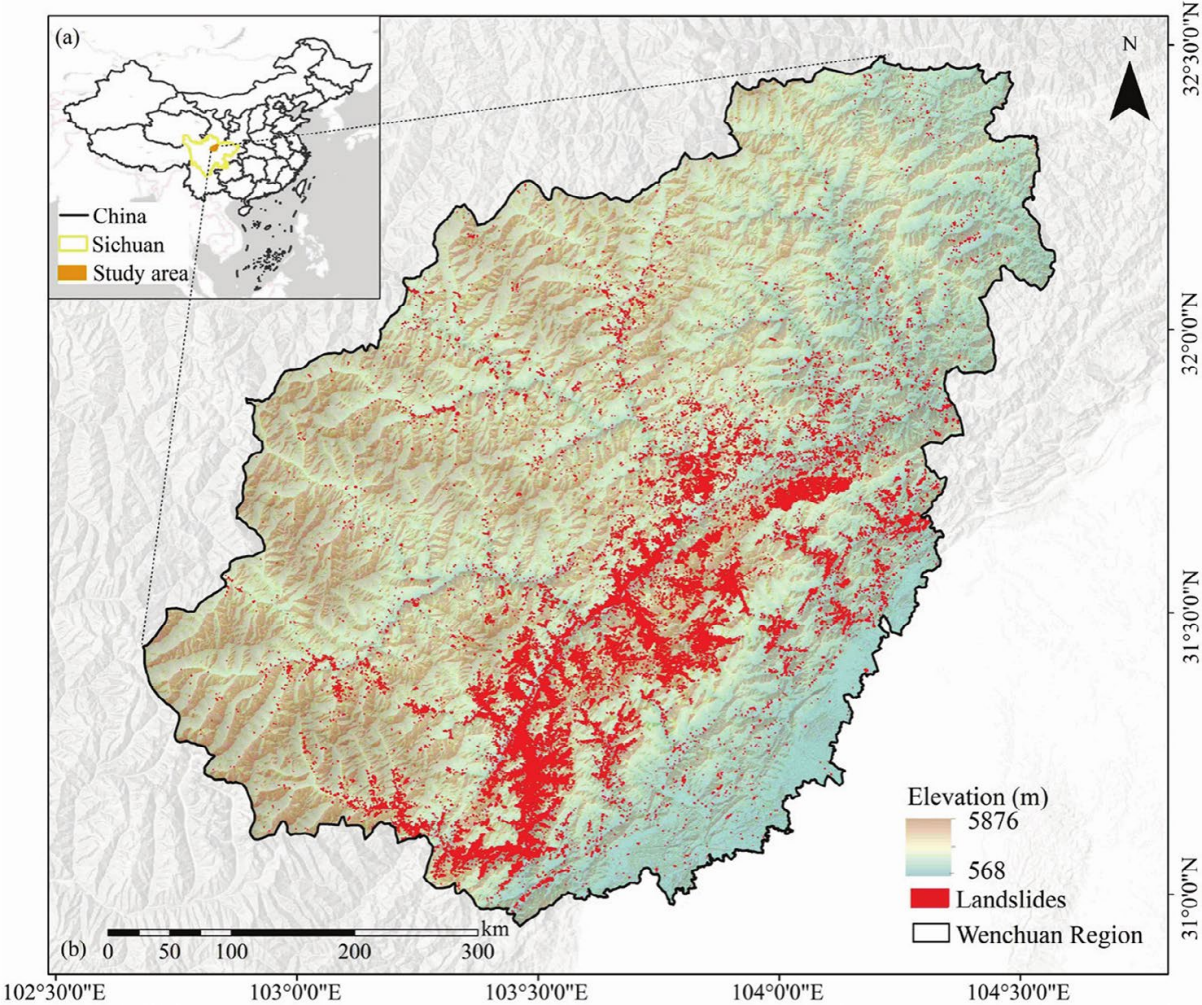
Overview of the study area. (a) Location of the study area in China. (b) Spatial distribution of co‐seismic landslides triggered by the 2008 Wenchuan earthquake.

The catastrophic 2008 Mw 7.9 Wenchuan earthquake profoundly destabilized the Longmenshan fault zone, triggering extensive co‐seismic landslides that drastically altered regional geo‐ecological conditions (Fan et al. 2019; Xu et al. 2009). This study focuses on the central segment of the fault zone (31.00°–31.90° N, 103.00°–104.00° E), encompassing the severely affected counties of Wenchuan, Beichuan, Lixian, and Maoxian (Figure 1). Located along the India–Eurasia collision front, the region is characterized by intense tectonics, high‐relief topography, and deeply incised valleys—features that collectively define a highly susceptible geohazard landscape. The earthquake‐induced shaking exploited these geological conditions to generate widespread slope failures, initiating long‐term landscape adjustment and ecosystem reorganization. These characteristics make the area an ideal natural laboratory for studying post‐seismic landslide cascades and vegetation recovery within active orogenic systems.

### 2.2 Datasets

#### 2.2.1 Vegetation Data

Vegetation dynamics were characterized using a 30‐m annual maximum NDVI dataset (2007–2023) obtained from the Geographic Data Sharing Infrastructure, Global Resources Data Cloud. The dataset was generated within the Google Earth Engine (GEE) platform based on Landsat 5 TM, Landsat 7 ETM+, Landsat 8 OLI, and Landsat 9 OLI‐2 Collection 2 Level‐2 surface reflectance products. According to the dataset documentation, multi‐source Landsat imagery was processed using a unified workflow including radiometric calibration, atmospheric correction, cloud masking, annual maximum NDVI compositing, and kernel‐based gap filling for Landsat 7 SLC‐off imagery to improve the temporal consistency of multi‐sensor observations.

#### 2.2.2 Topographic Data

To evaluate the impact of terrain on vegetation recovery, we used the ASTER GDEM V3 dataset, a global 30‐m resolution digital elevation model (DEM) produced by NASA's Terra satellite. The data were obtained from the Geospatial Data Cloud Platform. Based on this DEM, we extracted four terrain attributes relevant to vegetation recovery: elevation (ELE), slope (SLO), aspect (ASP), and terrain wetness index (TWI).

#### 2.2.3 Landslide Data

The co‐seismic landslide inventory was obtained from Wang et al. (2024) who produced it using the Continuous Change Detection and Classification (CCDC) algorithm applied to a time series of 521 Landsat images (2000–2021) on the GEE platform. CCDC models pixel‐level temporal trajectories and detects abrupt departures from long‐term spectral trends, enabling reliable extraction of co‐seismic landslides.

#### 2.2.4 Meteorological Data

Monthly temperature and precipitation data (2007–2020) at 1 km resolution were obtained from the National Earth System Science Data Center (Peng et al. 2019). These data were generated by downscaling the global 0.5° CRU dataset using the Delta method and validated against 496 meteorological stations; potential evapotranspiration was subsequently calculated using the Hargreaves equation. Surface solar radiation data (500 m, 2007–2020) were derived from the MODIS MCD18A1.061 product via Google Earth Engine (Wang 2021). Atmospheric humidity variables, including vapor pressure deficit and relative humidity, were extracted from the High‐resolution Atmospheric Humidity Index dataset for China (HiMIC‐Monthly), which has demonstrated high accuracy (*R* ^2^ > 0.96) (Zhang et al. 2024).

Based on these datasets, multi‐annual means of temperature (Temp), precipitation (Prec), potential evapotranspiration (ETp), solar radiation (DSSR), vapor pressure deficit (VPD), and relative humidity (RH) were calculated for 2007–2020 to represent the long‐term hydrothermal background conditions influencing post‐earthquake vegetation recovery. Specifically, precipitation and RH represent ambient moisture availability, VPD reflects atmospheric evaporative demand and plant water stress, whereas ETp integrates the combined effects of temperature and radiation on potential water loss (Allen et al. 1998; Novick et al. 2016). To ensure spatial consistency with the 30 m landslide and vegetation layers, all datasets were resampled to the same grid size via bilinear interpolation.

## 3 Methodology

**FIGURE 2:**
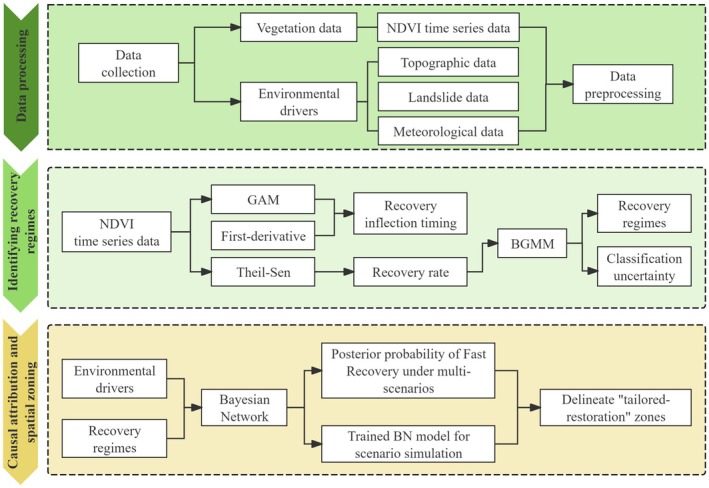
Analytical workflow of this study.

The methodological workflow consists of three phases (Figure 2). First, NDVI and environmental data were preprocessed. Second, recovery trajectories and regimes were identified using Generalized Additive Models (GAMs) and Bayesian Gaussian Mixture Model (BGMM). Finally, a BN was developed to evaluate recovery scenarios and delineate “tailored‐restoration” zones.

### 3.1 Trend Analysis of Post‐Landslide Vegetation Dynamics

Post‐seismic vegetation dynamics within landslide areas were evaluated at the pixel level by extracting NDVI time series using landslide masks. To obtain robust estimates of long‐term trends under the noisy and often non‐normal conditions of mountainous terrain, we applied the nonparametric Theil–Sen estimator to quantify NDVI slope changes and assessed their significance using the Mann–Kendall test (Gocic and Trajkovic 2013; Kendall 1948; Sen 1968). These nonparametric techniques are resistant to outliers and do not rely on distributional assumptions, providing a reliable baseline characterization of post‐seismic vegetation trajectories prior to subsequent nonlinear dynamic analyses.

### 3.2 Nonlinear Dynamic Modeling of Vegetation Recovery Processes

Post‐seismic recovery is a nonlinear process in which variations in recovery dynamics reflect ecosystem resilience (Liu et al. 2017). To capture continuous nonlinear trajectories without imposing predefined functional forms, we applied pixel‐wise GAMs with smoothing splines implemented via the pygam library (version 0.9.1) in Python. GAMs are particularly suitable for characterizing continuous, multi‐phase ecological recovery processes compared with parametric models or abrupt change‐detection approaches (e.g., BFAST) (Arij et al. 2026). Smoothing parameters were optimized using generalized cross‐validation (GCV) to balance model flexibility and overfitting (Wood 2020; Yang et al. 2003). For each fitted trajectory, the first derivative was calculated to identify the recovery inflection point, defined as the timing of the maximum instantaneous growth rate marking the transition from accelerated to decelerated regrowth. This inflection point serves as a trajectory‐based reference for delineating the fast‐regrowth phase.

To obtain a robust and spatially comparable measure of ecosystem recovery performance, the recovery‐rate metric was quantified as the overall vegetation growth trend during the fast‐regrowth phase rather than the maximum instantaneous growth rate identified from the GAM derivative. Reflecting the cumulative nature of ecological recovery, a pixel‐adaptive fast‐regrowth phase was defined for each pixel, extending from the beginning of the valid NDVI trajectory to the trajectory‐derived recovery inflection point. The Theil–Sen estimator was then applied to all NDVI observations within this trajectory‐defined phase to derive the recovery‐rate metric (year^−1^), which subsequently served as the input variable for the BGMM classification and BN analyses. Pixels with inflection points near the beginning or end of the observation period were treated separately: if the overall NDVI trend remained positive, the recovery rate was estimated from the full valid time series; otherwise, the pixel was excluded. Sensitivity analyses confirmed that recovery‐rate estimates are robust across a range of temporal‐window configurations, with mean rates and standard deviations largely consistent (Table S1), demonstrating that the metric reliably captures vegetation recovery while remaining insensitive to minor window variations.

### 3.3 Identification of Vegetation Recovery Regimes

To identify vegetation recovery regimes, we applied a Variational BGMM to the pixel‐level fast‐phase recovery rates. Unlike deterministic clustering methods (e.g., k‐means) or conventional Gaussian mixture models, the Variational Bayesian formulation estimates posterior distributions of model parameters and provides probabilistic cluster assignments, allowing uncertainty in transitional recovery states to be quantified while reducing the risk of overfitting (Murphy 2012).

Because recovery regimes represent both statistical patterns of recovery‐rate distributions and ecological interpretations of recovery capacity, the number of regimes was not determined solely by clustering outputs. Alternative BGMM configurations, including two‐ and three‐component solutions, were evaluated based on their statistical representation, interpretability, and relevance to subsequent ecological analyses. The final classification was selected as an analytical framework for distinguishing contrasting recovery capacities rather than as an assumption of discrete ecological states.

In the BGMM framework, the recovery rate *r* of each pixel is represented as a mixture of *K* Gaussian components (Bishop and Nasrabadi 2006):

$$p\left(r|\pi ,\mu ,\sigma^{2}\right)=\sum_{k=1}^{K}\pi_{k}N\left(r|\mu_{k},\sigma_{k}^{2}\right)$$

where $\pi_{k}$ is the mixing weight of the *k*th recovery component ($\sum \pi_{k}=1$), and $N\left(r|\mu_{k},\sigma_{k}^{2}\right)$ denotes a normal distribution with mean $\mu_{k}$ and variance $\sigma_{k}^{2}$.

The analysis was implemented using the BayesianGaussianMixture class in the scikit‐learn package (version 1.6.1) (Pedregosa et al. 2011). For each pixel, the model returned posterior membership probabilities and the corresponding cluster assignment. Posterior probabilities were further retained to quantify classification confidence and identify transitional recovery zones.

### 3.4 Bayesian Network‐Based Analysis of Vegetation Recovery Drivers and Management Zoning

#### 3.4.1 Screening of Driving Drivers and Multicollinearity Diagnosis

To construct a BN with high robustness and interpretability, minimizing multicollinearity among environmental drivers is critical for accurate parameter estimation and causal inference. Informed by prior research on post‐earthquake vegetation dynamics (Chen et al. 2022; Jiao et al. 2014; Mao et al. 2024; Pourghasemi et al. 2012; Wang, Yue, et al. 2022), we initially identified twelve candidate variables spanning topographic, climatic, and surface‐condition domains (e.g., ELE, SLO, Prec, Temp, and pre‐post disturbance NDVI difference [ΔNDVI]).

We first evaluated linear associations using a Pearson correlation matrix, followed by the Variance Inflation Factor (VIF) as the primary diagnostic metric, setting a threshold of VIF > 5 for exclusion. The variable selection followed an iterative procedure balancing statistical rigor with ecological reasoning (Austin 2002): among highly collinear variables, priority was given to those with clearer mechanistic links to vegetation recovery. Specifically, when multiple variables exceeded the threshold, the one with the highest VIF and lower ecological relevance was sequentially removed. This strategy ensured the retention of key predictors while effectively minimizing redundancy.

#### 3.4.2 Bayesian Network Model Construction

To characterize the nonlinear and probabilistic relationships between vegetation recovery and its environmental drivers, we applied a BN framework. As a probabilistic graphical model, a BN encodes dependency structures among variables through a directed acyclic graph (DAG) and quantifies these dependencies via conditional probability tables (CPTs) (Marcot et al. 2006; Pearl 2014; Shi et al. 2024). The DAG topology was specified a priori based on expert knowledge and current ecological understanding of post‐earthquake vegetation recovery processes. Directed links among topographic, climatic, vegetation‐condition, and recovery variables were defined according to established ecological mechanisms and evidence from previous studies (Hu et al. 2024; Wang et al. 2024; Cheng et al. 2025). According to the chain rule, the joint distribution of all variables is factorized as follows:

$$P\left(X_{1},X_{2},\ldots ,X_{n}\right)=\prod_{i=1}^{n}P\left(X_{i}|\text{parents}\left(X_{i}\right)\right)$$

where $\text{parents}\left(X_{i}\right)$ denotes the parent set of node $X_{i}$.

The BN constructed here consists of one target node (recovery rate) and nine environmental driver nodes. To capture nonlinear ecological responses and facilitate probabilistic inference, all continuous variables were discretized into three states (Low, Medium, High) using the Natural Breaks (Jenks) method (Jenks 1967) (Table 1). Pixel‐level values extracted from the discretized raster layers were subsequently used to estimate the CPTs. This framework provides a probabilistic representation of vegetation recovery under varying environmental conditions, enabling inference of the mechanisms governing post‐disturbance recovery dynamics (Dang et al. 2025).

**TABLE 1:** Description of the discretized BN nodes with low, medium, and high states.

Node	States and ranges	Unit
NDVI_pre	Low (< 0.58), medium (0.58–0.77), high (0.77–1.00)	—
ΔNDVI	Low (< 0.11), medium (0.11–0.25), high (0.25–0.83)	—
DEM	Low (< 1959.17), medium (1959.17–2854.02), high (2854.02–4913.85)	m
ASP	Cold (< −0.33), neutral (−0.33–0.45), warm (0.45–1.00)	—
TWI	Low (< 5.51), medium (5.51–11.29), high (11.29–31.17)	—
Temp	Low (< 5.86), medium (5.86–10.66), high (10.66–16.80)	°C
Prec	Low (< 854.54), medium (854.54–919.27), high (919.27–1094.28)	mm
VPD	Low (< 3.28), medium (3.28–4.23), high (4.23–7.66)	hPa
DSSR	Low (< 417.71), medium (417.71–479.02), high (479.02–613.65)	W/m^2^

*Note:* ASP is the cosine‐transformed aspect index (−1 to 1); higher values indicate warmer, south‐facing slopes.

#### 3.4.3 Model Performance Evaluation and Key Driver Identification

Rigorous evaluation is a prerequisite for reliable ecological inference using BN. We randomly partitioned the dataset into a training set (80%) and an independent testing set (20%) for model training and validation, respectively. To further assess the potential influence of spatial autocorrelation, an additional spatial block cross‐validation analysis was conducted using block sizes ranging from 10 × 10 to 200 × 200 pixels. Entire blocks were assigned to the training and testing subsets to reduce spatial leakage, and model performance was compared across different block scales. Model performance was comprehensively assessed using three metrics computed in Netica (version 5.18; Norsys Software Corp., Vancouver, Canada): Error Rate, Quadratic Loss, and Spherical Payoff. Error Rate measures overall classification accuracy (Hastie et al. 2009). Quadratic Loss evaluates the reliability of probabilistic predictions by penalizing confident errors (Guo et al. 2017), and is calculated as:

$$L=\frac{1}{N}\sum_{i=1}^{N}\sum_{J=1}^{C}{\left(p_{\mathrm{ij}}-o_{\mathrm{ij}}\right)}^{2}$$

where *N* is the number of test samples, *C* is the number of target classes, $p_{\mathrm{ij}}$ is the predicted probability that sample *i* belongs to class *j*, and $o_{\mathrm{ij}}$ is an indicator variable taking the value 1 if the true class of sample *i* is *j*, and 0 otherwise.

Spherical Payoff assesses the model's ability to assign high probabilities to the correct outcomes, with values closer to 1 indicating superior performance (Gneiting and Raftery 2007). It is calculated as follows:

$$S=\frac{1}{N}\sum_{i=1}^{N}\frac{p_{\mathrm{ic}}}{\sqrt{\sum_{j=1}^{C}{p_{\mathrm{ij}}}^{2}}}$$

where $p_{\mathrm{ic}}$ is the predicted probability for the true class *c* of sample *i*.

To identify key drivers of vegetation recovery regimes, we performed a sensitivity analysis using Variance Reduction (VR). This method quantifies a driver's influence by measuring the reduction in the target node's variance when the driver's state is known (Soboĺ 1993). Higher VR values indicate stronger explanatory power. The VR metric is defined as:

$$\mathrm{VR}\left(Q\right)=\mathrm{Var}\left(T\right)-\sum_{q}P\left(q\right)\cdot \mathrm{Var}\left(T|Q=q\right)$$

where *T* is the target variable, *Q* is a driver variable, $P\left(q\right)$ is the probability that *Q* takes state *q*, $\mathrm{Var}\left(T\right)$ is the total variance of *T*, and $\mathrm{Var}\left(T|Q=q\right)$ is the conditional variance given *Q* = *q*.

#### 3.4.4 Identification of Vegetation Recovery Regimes and Spatial Zoning Optimization

To reveal the environmental constraints shaping divergent vegetation recovery regimes and to delineate management‐relevant intervention pathways, we conducted diagnostic reasoning and scenario analysis using the validated BN (Lloyd et al. 2024). In the diagnostic phase, the target node “Recovery Rate” was fixed to “Fast” and “Slow” (100% each), and posterior driver distributions were inferred to characterize conditions associated with contrasting recovery regimes. To focus on actionable levers, we excluded two baseline drivers and retained the top three controllable drivers identified by sensitivity analysis.

We then performed predictive scenario analysis to evaluate how modifications to key drivers shift the posterior probability of Fast Recovery. Specifically, the net gain in Fast Recovery probability (Δ*P*_recovery, %) was calculated as follows:

$$\Delta P_{\text{recovery}}=P_{\text{post}}-P_{\mathrm{pre}}$$

where $P_{\mathrm{pre}}$ and $P_{\text{post}}$ represent the BN probabilities of the Fast Recovery state before and after scenario intervention, respectively.

Two complementary pathway‐identification strategies were implemented: Core Intervention Zones, defined as areas with otherwise favorable baseline conditions but at least one controllable driver in a limiting state; and Potential Enhancement Zones, characterized by suboptimal baseline conditions and at least one shared limiting driver. By evaluating each limiting driver individually under predefined baseline scenarios, we identified priority recovery pathways under varying environmental constraints, enabling differentiated ecological management strategies.

For visual representation of the identified pathways, we implemented the zoning rules in ArcGIS. We applied the specific state combinations to the previously discretized driver rasters, and pixels satisfying these conditions were extracted through conditional map‐algebra operations to generate the final zoning maps. The effectiveness of the zoning scheme was evaluated using the Mann–Whitney *U* test to quantify the significance of the “deficiency effect” (Mann and Whitney 1947), with effect sizes measured by Cohen's *d* (Cohen 2013). To control for Type I error inflation due to multiple comparisons, *p*‐values were adjusted using the False Discovery Rate (FDR) correction (Benjamini and Hochberg 1995). A consistency test was further applied to Core Intervention Zones to verify the stability of single‐driver intervention effects across different background conditions, thereby ensuring the robustness of management priorities.

## 4 Results

### 4.1 Spatio‐Temporal Patterns of Vegetation in Landslide‐Affected Areas

**FIGURE 3:**
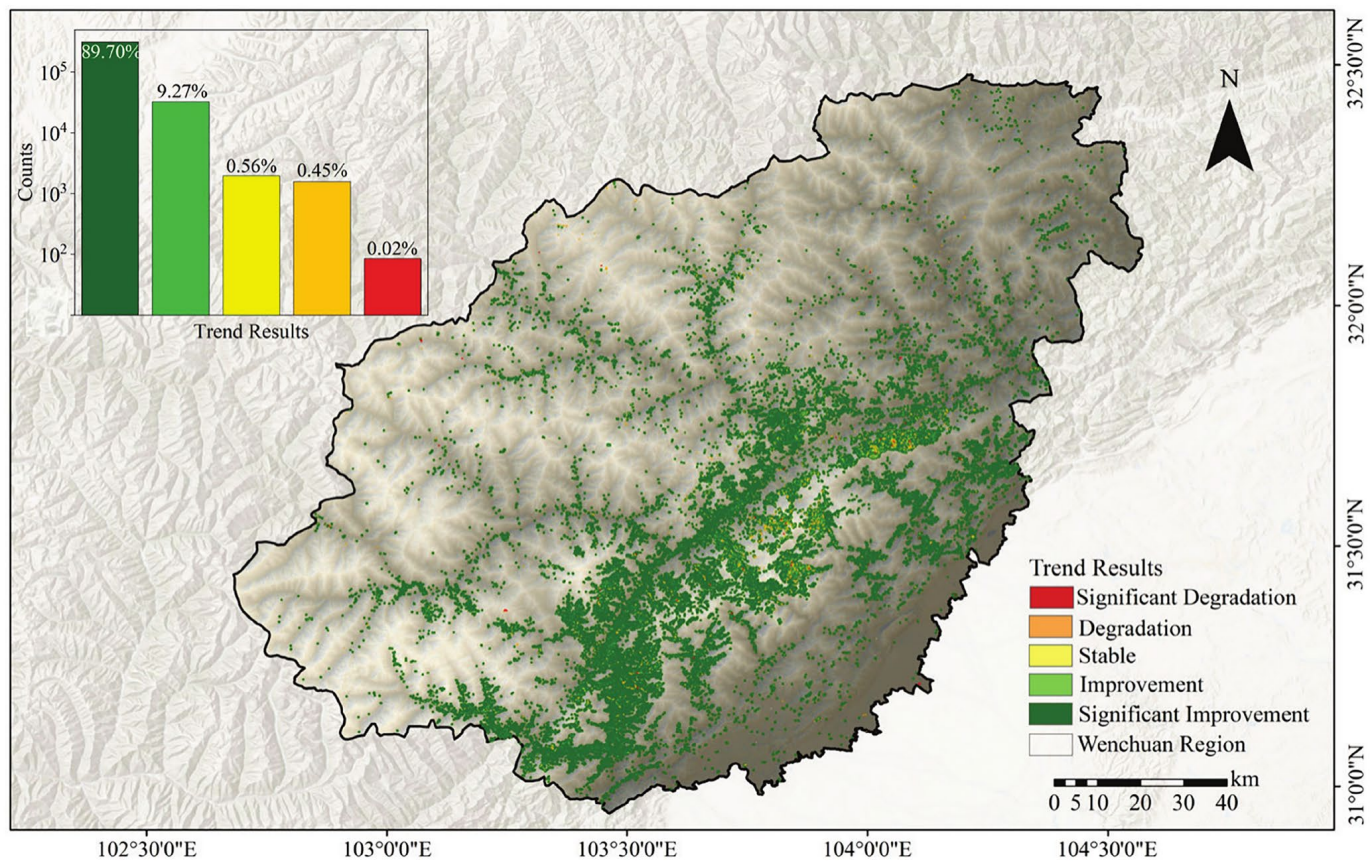
Spatial distribution of the long‐term NDVI trends within the mapped landslide inventory in the Wenchuan region (2008–2023).

Long‐term vegetation dynamics (2008–2023), assessed using the Theil–Sen estimator and Mann–Kendall test, revealed a pronounced overall greening trend within the mapped landslide inventory (Figure 3). The significant improvement category overwhelmingly dominated the NDVI trajectories, accounting for 89.70% of all analyzed pixels, whereas degradation and stable trends together represented less than 1.1% of the area.

Spatially, significant improvements were concentrated in the central region, forming a continuous southwest–northeast corridor of greening within the landslide inventory, whereas slight improvements were more diffusely distributed throughout the affected area. In contrast, degradation and stable trends were limited in extent and mainly clustered in the central‐eastern region. Significant degradation remained spatially restricted and highly fragmented across the landscape.

### 4.2 Nonlinear Recovery Dynamics: Inflection Timing and Recovery Rates of Post‐Landslide Vegetation

**FIGURE 4:**
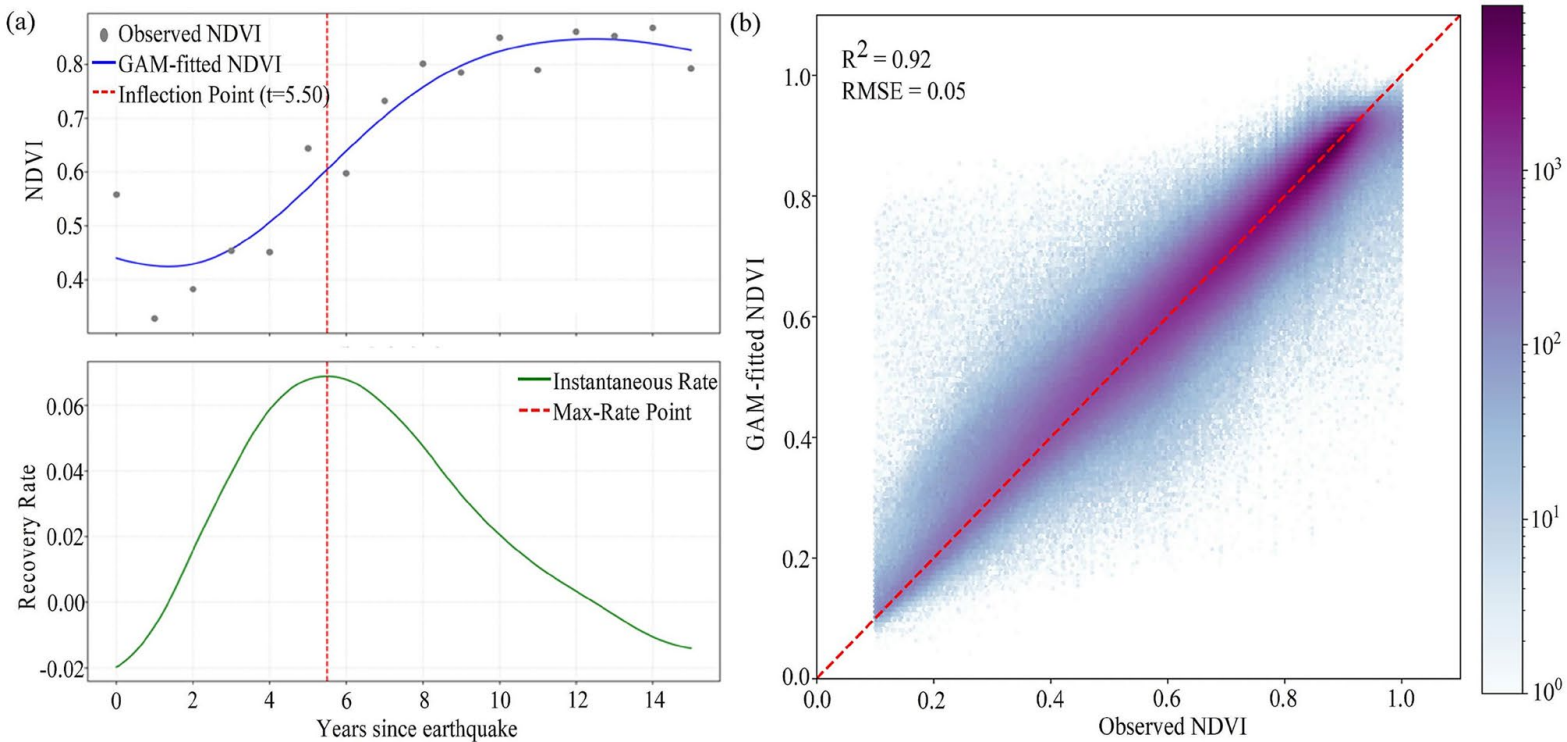
Illustration of vegetation recovery trajectory and inflection point identification derived from the GAM‐based first‐derivative approach. (a) Example of the GAM‐fitted NDVI recovery curve and its first‐derivative series. (b) Scatter plot of observed NDVI and GAM‐fitted NDVI values.

Post‐landslide vegetation recovery exhibited pronounced nonlinear trajectories. We characterized these dynamics by fitting GAMs to pixel‐level NDVI time series and computing first‐order derivatives to quantify instantaneous recovery rates (Figure 4a). The fitted models demonstrated high fidelity to the observational data (Figure 4b), with strong agreement between observed and GAM‐fitted NDVI values (*R* ^2^ = 0.92, RMSE = 0.05), thereby supporting the robustness of the derived recovery metrics, including recovery rates and inflection timing. The derived inflection point, defined as the timing of the maximum instantaneous growth rate (from the GAM derivative), represents a trajectory‐based transition from accelerated vegetation establishment to a subsequent phase of growth maturation and consolidation. For the representative pixel shown in Figure 4a, this inflection occurred approximately 5.5 years post‐disturbance. By identifying these dynamic features, the GAM–derivative framework provided a rigorous basis for the subsequent spatial analysis of recovery phases.

**FIGURE 5:**
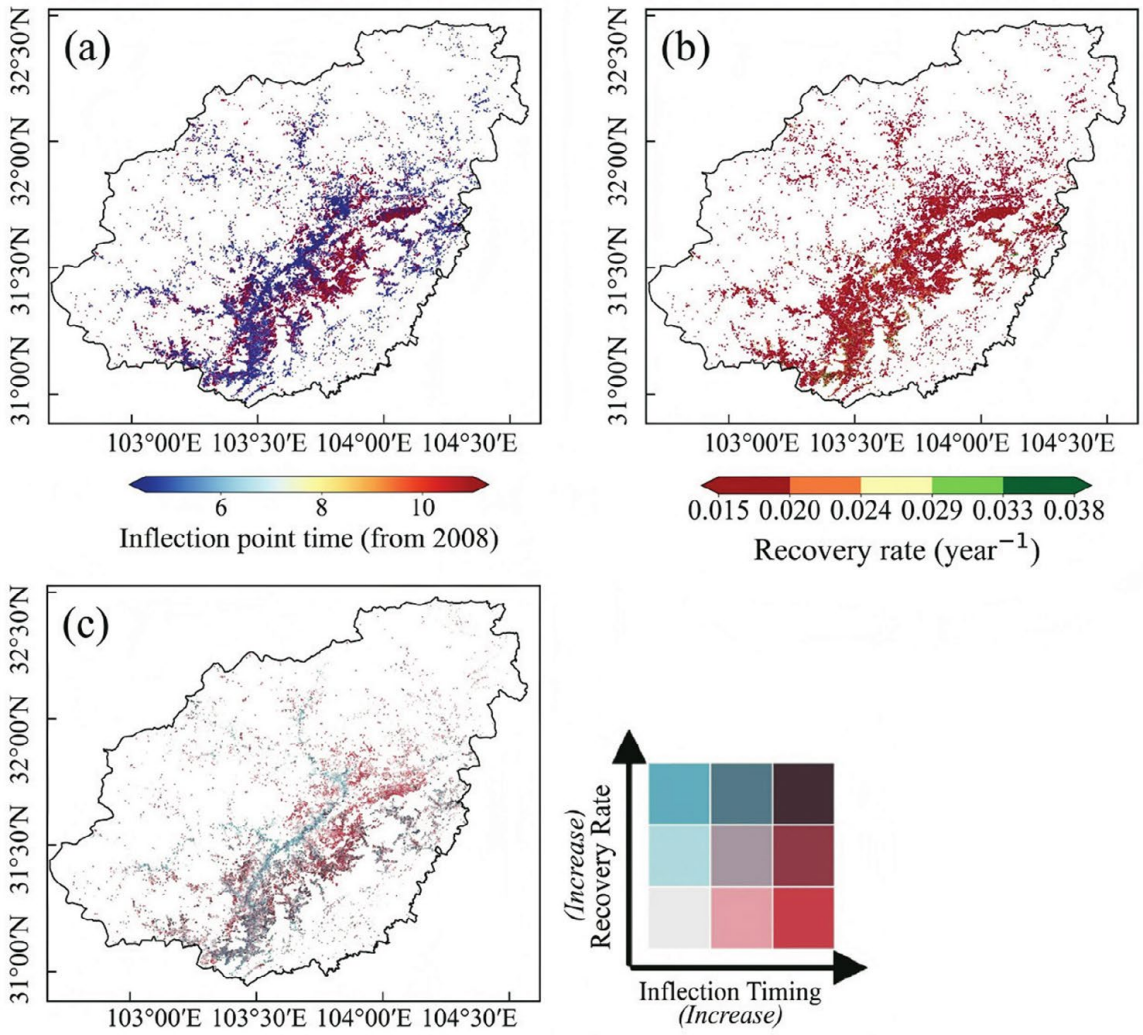
Mapping of vegetation recovery trajectories: (a) recovery inflection timing; (b) recovery rate estimated from the GAM‐derived NDVI trajectories; and (c) bivariate visualization showing the spatial covariation between recovery timing and recovery rate.

Spatially, the timing of recovery inflection points displayed distinct geographic patterns (Figure 5a). Most disturbed pixels exhibited inflection points within 5–7 years post‐earthquake, indicating a regionally consistent period of accelerated recovery. In contrast, substantially delayed inflection points (> 10 years) were concentrated in high‐elevation areas in the northern, northeastern, and southeastern subregions, where steep terrain and harsher environmental conditions likely constrained vegetation establishment and ecosystem stabilization. Recovery rates also showed substantial spatial heterogeneity (Figure 5b). Most pixels exhibited moderate recovery rates (0.015–0.025 year^−1^), whereas higher recovery rates (> 0.03 year^−1^) were primarily concentrated in low‐elevation valleys characterized by more favorable hydrothermal conditions. Lower recovery rates were generally associated with steeper slopes, higher elevations, and moisture‐limited environments.

To better visualize the spatial covariation between recovery timing and recovery rate, a bivariate map was further constructed (Figure 5c). The combined patterns reveal a clear spatial divergence in post‐seismic recovery trajectories. Areas exhibiting early inflection timing and rapid recovery rates were mainly distributed in valley regions and lower‐elevation environments, reflecting relatively favorable ecological conditions for rapid vegetation re‐establishment. By contrast, regions characterized by delayed inflection timing and slower recovery rates were concentrated in mountainous areas with harsher topographic and hydrothermal constraints. Intermediate combinations of recovery timing and recovery rate formed transitional zones surrounding the major landslide corridors, suggesting strong spatial heterogeneity in ecosystem recovery processes.

Overall, these results indicate that post‐seismic vegetation recovery across the Wenchuan earthquake region was spatially widespread but highly uneven, with both recovery timing and recovery intensity strongly regulated by local environmental gradients.

### 4.3 Spatial Differentiation of Vegetation Recovery Regimes

**FIGURE 6:**
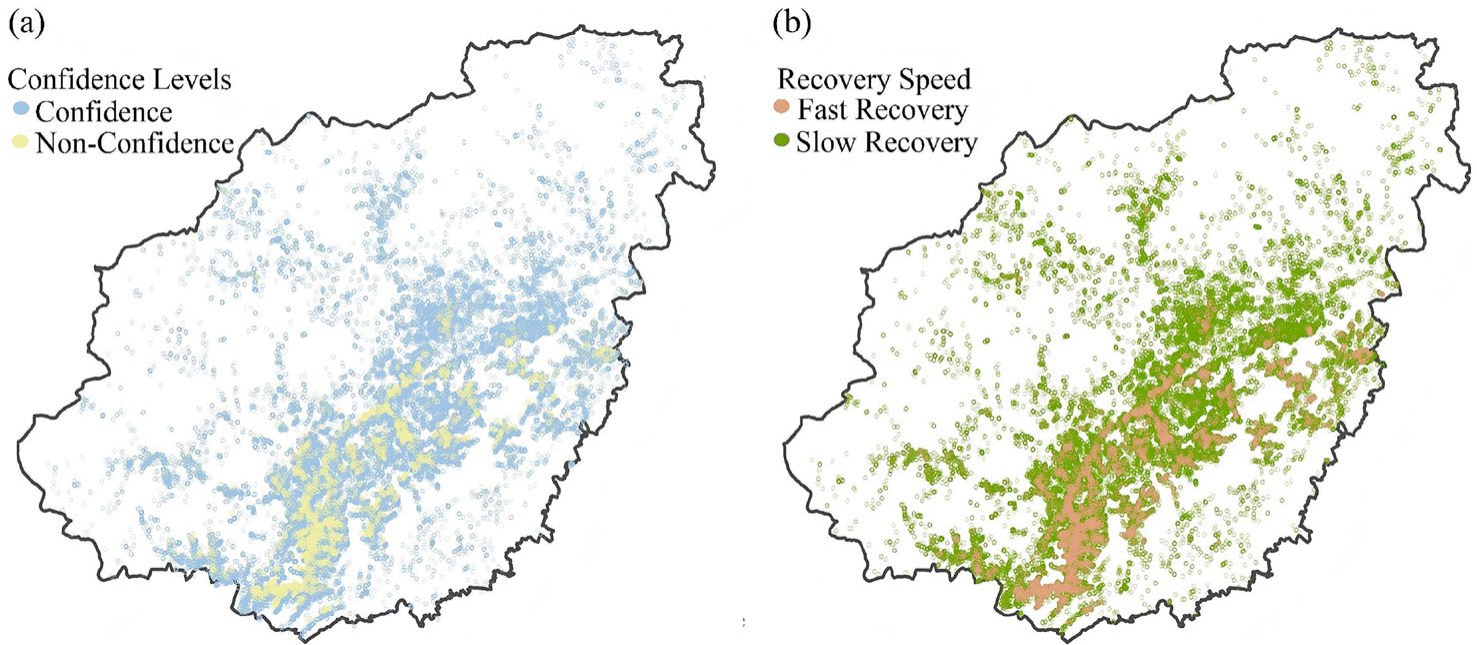
Spatial patterns of vegetation recovery regimes and BGMM‐derived confidence levels. (a) Spatial distribution of classification confidence levels derived from BGMM posterior probabilities. (b) BGMM‐based classification of vegetation recovery into “Slow Recovery” and “Fast Recovery” regimes.

Model confidence analysis showed that most pixels were assigned to recovery regimes with confidence (Figure 6a). Approximately 95% of pixels were assigned to recovery regimes with confidence, whereas only ~5% were classified as non‐confidence pixels. These non‐confidence pixels were primarily concentrated along regime boundaries and ecological transition zones, reflecting gradual transitions in recovery‐rate characteristics rather than instability of the classification model. The confidence levels showed no apparent spatial correspondence with the recovery regime classification, indicating that confidence assessment and recovery regime identification represent complementary but distinct outputs of the BGMM framework.

Based on the BGMM classification, two contrasting vegetation recovery regimes were identified from the continuous distribution of post‐seismic recovery rates (Figure 6b). The Slow Recovery regime exhibited lower mean recovery rates and dominated the study area, accounting for 92.12% of classified pixels. In contrast, the Fast Recovery regime showed substantially higher recovery rates but represented only 7.88% of classified pixels, mainly clustering along river valleys and low‐elevation areas in the central–southern region. The two regimes displayed a fragmented spatial mosaic, particularly across areas with abrupt topographic transitions, highlighting the strong influence of local terrain and hydrothermal conditions on vegetation recovery patterns.

Additional evaluation using a three‐component BGMM revealed an alternative Slow–Medium–Fast representation of the recovery‐rate distribution. Although the K = 3 solution provided a finer statistical partition of the continuous recovery‐rate gradient, the intermediate Medium Recovery component mainly represented transitional variation within the recovery continuum rather than a clearly distinguishable recovery pathway. Therefore, the two‐component classification was retained as a parsimonious representation of contrasting recovery capacities for subsequent analyses.

### 4.4 Bayesian Network‐Based Analysis of Recovery Mechanisms and Spatial Optimization

We employed a BN framework to diagnose the probabilistic drivers shaping post‐seismic vegetation recovery regimes. This analysis quantified the interactions among key environmental controls, explicitly mapping the causal pathways underlying recovery patterns and revealing the spatial heterogeneity of intervention potential under varying ecological baselines.

#### 4.4.1 Driver Screening and Network Structural Characteristics

Multicollinearity diagnostics identified a subset of ecologically meaningful and statistically independent predictors (Figure S1). The final driver set consists of nine variables—pre‐earthquake NDVI (NDVI_Pre), ΔNDVI, Prec, Temp, DSSR, SLO, ASP, TWI, and VPD. All retained variables exhibited consistently low VIF values, indicating that this set provides a robust basis for modeling vegetation recovery regimes.

**FIGURE 7:**
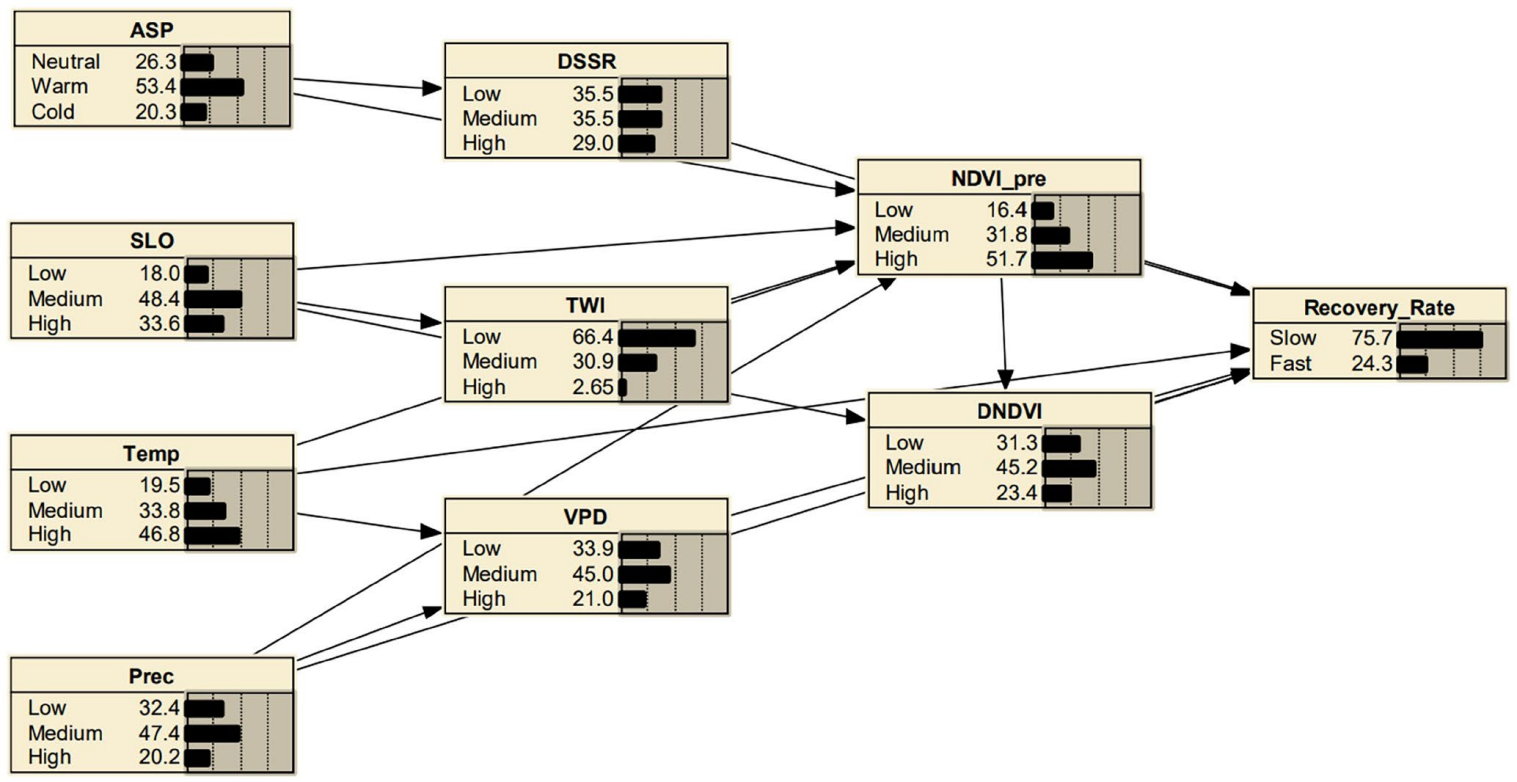
Structure of the trained BN for predicting vegetation recovery regimes. ΔNDVI is displayed as “DNDVI” in Netica.

The learned BN topology reveals a distinct hierarchical structure governing the formation of recovery regimes (Figure 7). Direct control over the recovery process is primarily exerted by ΔNDVI and NDVI_Pre. In contrast, climatic drivers (e.g., Prec, Temp) and topographic attributes (e.g., SLO, ASP) function as distal drivers, influencing the recovery outcome indirectly by regulating intermediate environmental conditions such as TWI and DSSR. This multi‐layered architecture highlights that post‐seismic recovery does not result from any single driver but emerges from the interplay between disturbance severity, baseline ecosystem properties, and local hydrothermal–topographic constraints. The conditional probability tables derived from parameter learning quantitatively capture these dependencies, providing an interpretable probabilistic basis for subsequent sensitivity analysis, diagnostic reasoning, and scenario‐based spatial optimization.

#### 4.4.2 Model Performance Validation and Identification of Key Drivers

The validated BN exhibits strong predictive performance, achieving an overall classification accuracy of 90.61%. Proper scoring‐rule metrics further corroborate the reliability of its probabilistic outputs, with a low logarithmic loss (0.29), a minimal quadratic loss (0.17), and a high spherical payoff (0.92). These results confirm that the model provides both accurate regime classifications and well‐calibrated probability estimates.

To assess spatial autocorrelation and leakage, we conducted multi‐scale spatial block cross‐validation (Table S2). The spatially constrained results were highly consistent with the original random‐split validation, maintaining high classification accuracy (89.58%–92.77%). Quadratic loss and logarithmic loss also showed minimal variation under increasing spatial separation between training and testing samples. These findings suggest that the predictive performance of the BN was not substantially affected by local spatial dependence, but instead reflect robust relationships between environmental conditions and vegetation recovery regimes.

Sensitivity analysis reveals a clear hierarchical structure in the drivers shaping recovery regimes (Table 2). ΔNDVI and NDVI_Pre emerge as the dominant predictors, together accounting for the largest share of explained variance (3.29% and 1.09%, respectively), identifying them as the primary inherent controls. Beyond these baseline constraints, several climatic variables emerged as critical determinants. DSSR (1.67%), Prec (1.66%), and Temp (1.13%) show contributions comparable to or exceeding that of NDVI_Pre, highlighting the dominant role of coupled hydrothermal–radiative conditions in driving spatial differentiation in recovery regimes.

**TABLE 2:** Results of the sensitivity analysis for the BN model.

Rank	Node	Percent of beliefs %	Variance of beliefs %
1	ΔNDVI	2.94	3.29
2	DSSR	1.5	1.67
3	Prec	1.42	1.66
4	Temp	1.06	1.13
5	NDVI_Pre	0.97	1.09
6	VPD	0.27	0.3
7	ASP	0.06	0.07
8	SLO	0.01	0.02
9	TWI	0.0009	0.001

#### 4.4.3 Environmental Pathways Differentiating Vegetation Recovery Regimes

**FIGURE 8:**
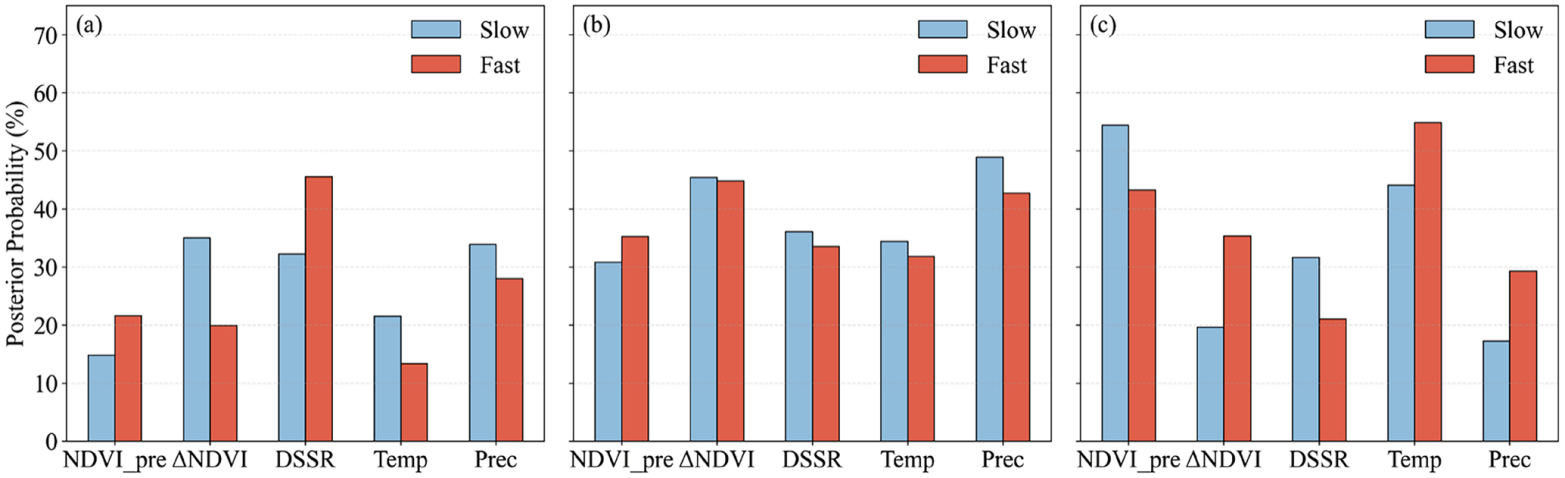
Contribution of environmental drivers to Slow and Fast vegetation recovery across state levels. (a) Low state, (b) Medium state, and (c) High state.

Distinct differences in the configurations of key variables between Slow and Fast recovery regimes are revealed across state levels (Figure 8), reflecting distinct hydrothermal and radiative patterns. At the low state (Figure 8a), regime differentiation is primarily expressed through limiting hydrothermal and radiative conditions. The Slow regime displays higher posterior probabilities of low Prec (33.90%) and low Temp (21.50%) than the Fast regime, reflecting stronger exposure to combined moisture and thermal constraints. In contrast, the Fast regime is characterized by a higher probability of low DSSR (45.50%), indicating an association between rapid recovery and reduced radiative input under low‐state conditions.

Transitioning to the Medium state (Figure 8b), the two recovery regimes converge in their Prec responses, with peak probabilities at moderate Prec levels. In contrast, subtle divergences emerge in DSSR and Temp configurations. The Slow regime maintains higher posterior probabilities at the medium level (36.10% for DSSR and 34.40% for Temp), whereas the Fast regime shows slightly lower probabilities (33.50% and 31.80%, respectively), reflecting a weaker concentration within the medium range.

Regime differentiation becomes most distinct under the High state (Figure 8c). The Fast regime concentrates strongly at high Temp (54.90%) and high Prec (29.30%), with posterior probabilities exceeding those of the Slow regime. By comparison, the Slow regime exhibits a higher posterior probability at the high DSSR level (31.60%) than the Fast regime.

Overall, the analysis demonstrates that the Slow and Fast recovery regimes are associated with distinct combinations of hydrothermal and radiative conditions. Slow recovery is more frequently associated with colder and drier configurations accompanied by relatively higher radiative exposure, whereas Fast recovery tends to align with warmer and wetter conditions under comparatively moderate DSSR levels.

#### 4.4.4 Spatial Heterogeneity of Scenario‐Driven Recovery Potential

**FIGURE 9:**
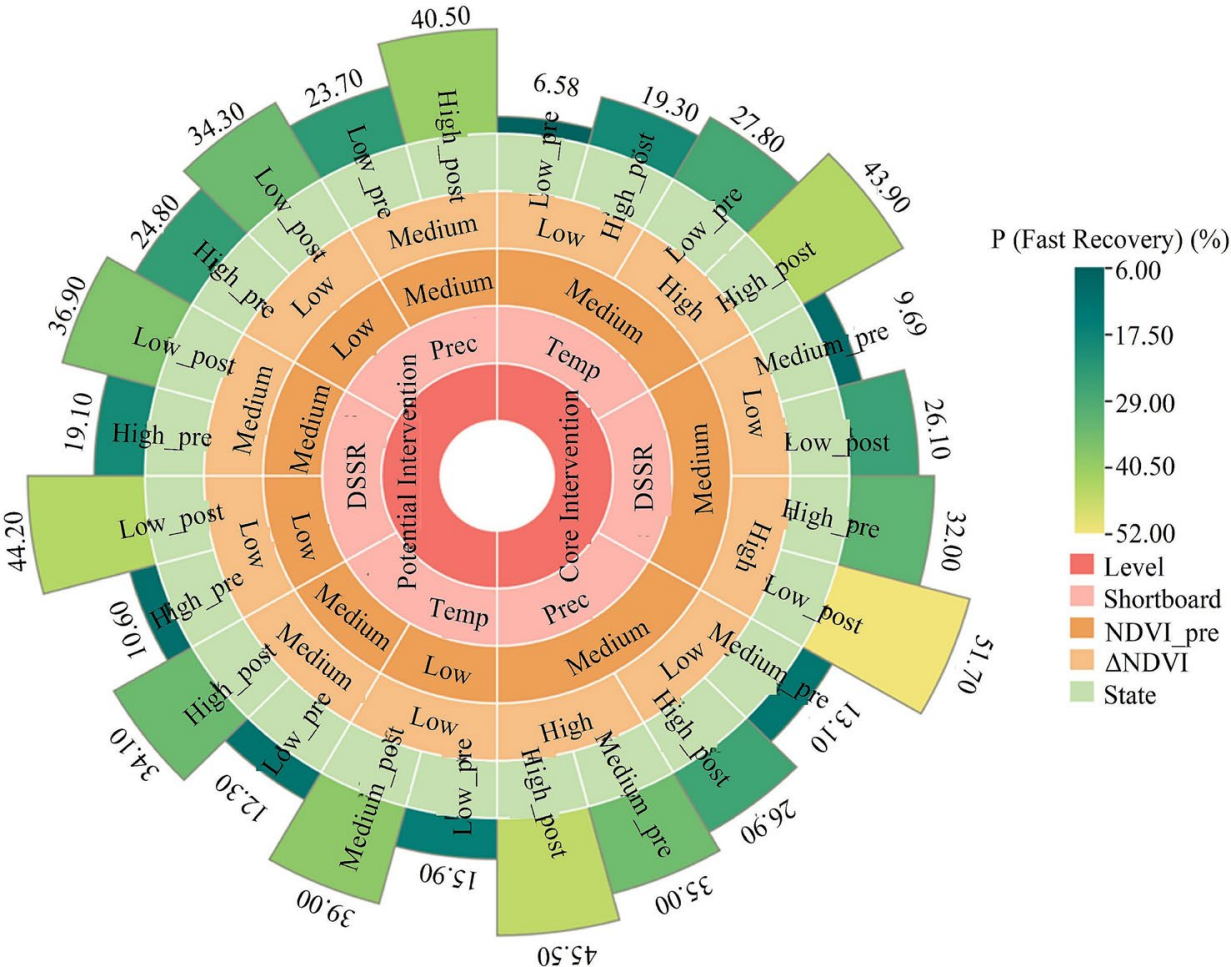
Scenario‐based gains in Fast Recovery probability across ecological baselines from alleviating limiting drivers (Prec, DSSR, and Temp). The underlying prior probabilities (*P*_pre_), posterior probabilities (*P*_post_), and Δ*P*_recovery_ values used to generate this figure are provided in Table S3.

Scenario‐based simulations utilizing the BN quantified how the alleviation of key constraints enhances recovery potential across distinct ecological baselines (Figure 9; Table S3). In the Core Intervention Zones, intervention efficacy exhibited strong baseline dependence. Under conditions of moderate vegetation cover and low disturbance (NDVI_Pre = Medium, ΔNDVI = Low), mitigating DSSR limitations yielded the most substantial increase in the probability of Fast Recovery (+16.41%), surpassing improvements derived from Prec (+13.80%) and Temp (+12.72%). Notably, as disturbance intensity increased (NDVI_Pre = Medium, ΔNDVI = High), the overall responsiveness of the system amplified, yet DSSR retained its dominance. The net improvement from DSSR mitigation (+19.70%) significantly exceeded that of Temp (+16.10%) or Prec (+10.50%), reaffirming energy availability as the primary bottleneck in these disturbance‐prone areas.

In Potential Enhancement Zones, the hierarchy of limiting drivers shifted markedly with baseline conditions. Under low vegetation cover and minimal disturbance (NDVI_Pre = Low, ΔNDVI = Low), alleviating DSSR constraints produced the largest increase in Fast Recovery probability (+33.60%), far exceeding the effects of Temp (+23.10%) and Prec (+9.50%), highlighting the dominance of energy limitation in low‐productivity systems. Once both vegetation and disturbance reached moderate levels (NDVI_Pre = Medium, ΔNDVI = Medium), the primary constraint transitioned: Temp emerged as the most influential driver (+21.80%), overtaking DSSR (+17.80%) and Prec (+16.80%), indicating a stage‐dependent transition from energy supply to thermal regulation of growth efficiency.

**FIGURE 10:**
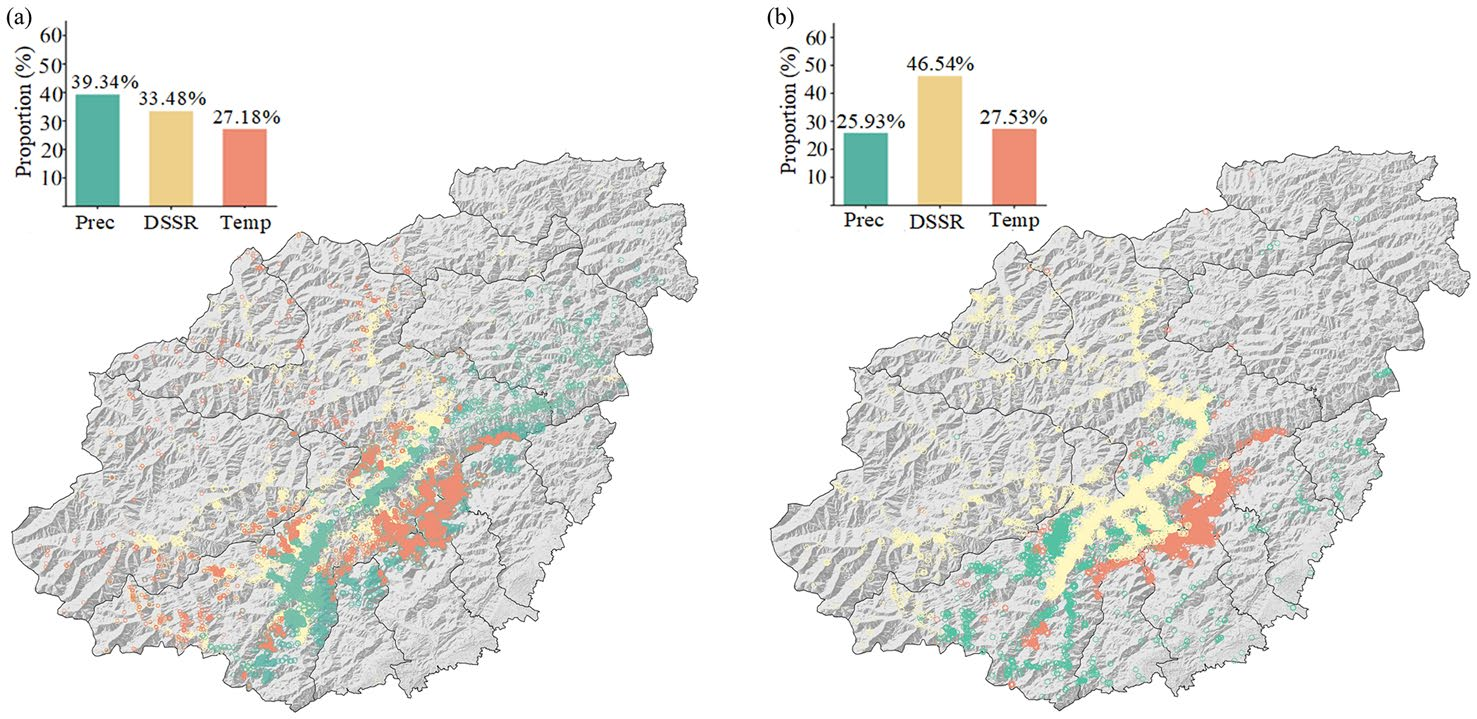
Spatial patterns of dominant limiting drivers for vegetation recovery. (a) Core Intervention Zones. (b) Potential Enhancement Zones.

Spatial mapping revealed distinct geographical contrasts in limiting drivers (Figure 10a,b). In Core Intervention Zones, Prec was the dominant constraint (39.34%), concentrated in downstream valleys and low‐elevation foothills bordering the Sichuan Basin, where local climate and human pressures likely limit water availability. Conversely, in Potential Enhancement Zones, DSSR became the prevailing limitation (46.54%), concentrated in high‐elevation ridges and deeply incised valleys. This spatial shift corroborates the scenario analyses: differing ecological baselines not only alter intervention effectiveness but also redistribute limiting constraints across the landscape, providing spatially explicit guidance for targeted restoration in the Wenchuan earthquake region.

## 5 Discussion

### 5.1 Ecological Significance of a Critical Recovery Window in Post‐Landslide Ecosystems

Our analysis verifies the pronounced nonlinearity of post‐disturbance recovery, with GAM‐fitted trajectories robustly identifying the timing of peak recovery rates. The strong spatial coherence of these inflection points delineates a critical 5–7‐year “window of opportunity” across much of the landscape (Figure 4a). This region‐wide pulse of accelerated regrowth suggests that ecosystems are capitalizing on released resources and newly opened niche space during the initial stabilization period. Ecologically, this inflection likely marks a pivotal transition: as physical substrate instability subsides, recovery becomes increasingly regulated by resource availability (e.g., water) and by biotic processes that reinforce early establishment (Hobbs and Harris 2001).

This temporal convergence suggests that internal ecological processes—such as nutrient stabilization, root‐network expansion, and facilitative interactions among pioneer species—play a more decisive role in determining recovery timing than previously recognized (Wardle et al. 2004). By contrast, areas with substantially delayed inflection points (> 10 years), concentrated in high‐elevation and topographically complex sectors, reveal a distinct “compound‐constraint” regime where steep slopes, microclimatic limitations, and seed‐dispersal barriers collectively suppress resilience (Chen et al. 2021; Körner and Kèorner 1999). These patterns suggest that recovery dynamics are governed not only by environmental gradients themselves, but also by their interactions with persistent disturbance legacies, including substrate instability, soil loss, and reduced vegetation cover. Together, these factors can substantially constrain vegetation establishment and slow ecosystem reorganization in environmentally harsh areas.

### 5.2 Bayesian Network Reveals Hierarchical Drivers, Optimal Configurations, and Spatially Explicit Interventions

The BN framework elucidated the hierarchical controls on vegetation recovery, revealing that the process is gated by baseline conditions rather than solely by climate. Network topology and sensitivity analysis confirmed that ΔNDVI and NDVI_Pre served as the primary constraints, collectively acting as a form of “ecological memory” that predisposed areas to Slow or Fast Recovery (Figure 7, Table 2).

Within this hierarchical structure, the BN posterior results indicate that transitions from Slow to Fast recovery are consistently associated with a specific environmental configuration combining favorable hydrothermal conditions with moderated radiative input. Fast recovery does not emerge in response to improvements in any single driver, but instead tends to occur only when sufficient moisture and elevated temperature coincide under non‐extreme radiation levels. This pattern explains why increasing precipitation alone produces limited recovery gains when thermal or radiative constraints persist. More broadly, the results reveal a state‐dependent and nonlinear structure in environmental contributions, with temperature becoming increasingly associated with Fast recovery only once hydrothermal and radiative conditions are jointly aligned, underscoring that efficient recovery reflects coordinated environmental conditions rather than the maximization of individual factors.

These state‐dependent relationships provide the basis for translating mechanism into management. Through scenario simulation, intervention efficacy proved highly baseline‐dependent. In Core Intervention Zones with moderate ecological conditions, mitigating DSSR stress was most effective. In Potential Enhancement Zones characterized by poorer initial states, a critical bottleneck shift occurred: DSSR limitation shifted to a Temp constraint as vegetation and disturbance levels improved, marking a transition from energy supply to growth efficiency controls. Spatialization of these relationships provided an actionable blueprint for restoration (Figure 10a,b). Prec limits dominated eastern lowlands, while DSSR formed a pervasive “highland bottleneck” in mid‐western ridges and shady slopes. This probabilistic spatial stratification enables a shift from uniform policy to precision ecology, offering a robust basis for targeting interventions to specific ecological and geographical contexts.

### 5.3 Limitations and Future Directions

Despite the strengths of the integrated framework, several limitations remain and offer valuable directions for future work. A key limitation arises from the temporal constraints of the Landsat time series: although the GAM‐based first‐derivative framework offers a robust means of characterizing recovery dynamics, the 16‐day revisit interval and cloud contamination limit the detection of short‐term variability in recovery rates. Higher‐frequency satellite data (e.g., Sentinel‐2, Harmonized Landsat–Sentinel) or fusion‐based time‐series reconstruction could enhance recovery‐rate estimation and enable more refined assessments of temporal variability in ecosystem resilience.

Another consideration is that the binary recovery classification should not be interpreted as the existence of two strictly discrete ecological states. Instead, it represents an ecological simplification of a continuous recovery‐rate spectrum, aiming to distinguish areas with contrasting recovery tendencies. Although a three‐component BGMM provided a finer statistical description of the recovery‐rate distribution, the intermediate Medium Recovery category represented transitional variation along the recovery continuum. Therefore, the Slow–Fast classification was adopted because it provides a more interpretable framework for summarizing dominant recovery patterns and identifying areas with contrasting recovery capacities while acknowledging the inherent continuum of ecosystem recovery processes.

Additionally, a minor temporal mismatch exists between the environmental predictors (averaged over 2007–2020 due to data availability) and the vegetation recovery trajectories extending to 2023. However, post‐seismic vegetation recovery represents a long‐term secondary succession process primarily constrained by persistent hydrothermal and radiative environmental gradients rather than short‐term climatic fluctuations (Palmer et al. 2016). Therefore, the multi‐annual climatic averages used in this study are considered sufficient to characterize the stable spatial heterogeneity of environmental conditions across the mountainous study area. Nevertheless, future studies integrating temporally synchronized climatic datasets may further improve understanding of the influence of short‐term extreme climate variability on vegetation recovery trajectories.

Furthermore, the BN's strength in probabilistic inference comes with inherent trade‐offs. The required discretization of continuous variables and the assumption of conditional independence simplify real‐world ecological feedbacks—particularly in transition zones where classification confidence is lowest (Figure 6a)—highlighting the need for frameworks such as Gaussian Process Networks or Structural Equation Modeling to better capture complex dependencies and strengthen causal inference.

Finally, while the scenario simulations effectively captured hydrothermal–radiative controls on recovery, the influence of anthropogenic drivers—such as ecological restoration programs, engineering stabilization, and land‐use change—was not explicitly incorporated. Future studies should integrate socioecological variables and explore how interactions between climatic variability and human interventions shape recovery potential under both current and projected climate conditions (Yan et al. 2024). Overall, addressing these limitations will broaden the explanatory power of post‐disturbance recovery models and improve their applicability for resilience assessment and adaptive ecosystem management in earthquake‐prone mountain environments.

## 6 Conclusion

Understanding the spatial patterns and drivers of post‐disturbance vegetation recovery is crucial for guiding targeted restoration in disaster‐prone mountain regions. By combining nonlinear trajectory analysis with Bayesian probabilistic inference, this study provides a transferable framework to capture both the temporal dynamics and environmental determinants of recovery across landslide‐affected areas after the 2008 Wenchuan earthquake. GAM analysis revealed that most areas reached a critical inflection point within 5–7 years, while delayed recovery (> 10 years) occurred mainly in high‐elevation and topographically complex subregions.

The BN revealed that vegetation recovery is shaped by the interaction between ecological memory and contemporary environmental conditions. NDVI_Pre and ΔNDVI together represent the ecological memory and disturbance footprint of the system, defining the initial conditions from which recovery proceeds. Against this inherited background, subsequent recovery trajectories are primarily regulated by the post‐landslide hydrothermal‐radiative environment. Fast recovery occurs under a “warm‐moist‐moderate light” configuration—adequate Prec, sufficient Temp, and moderated DSSR. Scenario‐based simulations further revealed spatially explicit limiting drivers: DSSR dominates in high‐elevation, rugged terrain, whereas Temp and Prec prevail in lower‐elevation or moderately disturbed areas. The hierarchy of constraints shifts with baseline conditions, indicating that interventions should be tailored—enhancing light to boost recovery in low‐productivity regions, and optimizing thermal and moisture conditions as vegetation stabilizes. Overall, this study offers both an integrated understanding of vegetation recovery regimes and a practical probabilistic framework for diagnosing ecosystem constraints. By linking recovery regimes to their causal drivers and spatial contexts, it provides actionable guidance for targeted restoration and a transferable approach for enhancing ecological resilience in mountainous regions facing increasing disturbances.

## Author Contributions

**Mingxuan Wan:** formal analysis (equal), investigation (equal), methodology (equal), validation (equal), visualization (equal), writing – original draft (equal). **Wei Zhao:** conceptualization (equal), funding acquisition (equal), project administration (equal), resources (equal), supervision (equal), writing – review and editing (equal). **Jiujiang Wu:** formal analysis (equal), writing – review and editing (equal). **Yanqing Yang:** investigation (equal). **Junli Zhao:** validation (equal), writing – review and editing (equal).

## Funding

This work was supported by the National Natural Science Foundation of China (U25A20769 and 42222109) and the Key Program of the Chinese Academy of Sciences for International Cooperation (162GJHZ2023065MI).

## Conflicts of Interest

The authors declare no conflicts of interest.

## Supporting information


**Table S1:** Sensitivity analysis of recovery‐rate estimates under alternative trajectory‐phase definitions.
**Figure S1:** Multicollinearity diagnostics for predictor variables. (a) Pearson correlation matrix for all initial variables. (b) VIF scores for the final selected variable set.
**Table S2:** Sensitivity analysis of Bayesian Network predictive performance across different spatial block cross‐validation scales.
**Table S3:** Derived prior and posterior probabilities of Fast Recovery under specific scenario interventions in the Bayesian Network.

## Data Availability

The representative Python scripts supporting trajectory analysis and recovery‐regime classification are archived in a reserved Zenodo repository (https://doi.org/10.5281/zenodo.20594642). All input datasets are publicly accessible: landslide inventory data are available at Zenodo (https://zenodo.org/records/10440752); vegetation index data (30‐m annual maximum NDVI dataset) are available from the Geographic Data Sharing Infrastructure, Global Resources Data Cloud (GIS5G) (https://www.gis5g.com); precipitation data at https://doi.org/10.5281/zenodo.3114194; temperature data at https://doi.org/10.5281/zenodo.3185722; and solar radiation data at https://doi.org/10.5067/MODIS/MCD18A1.062. Digital Elevation Model (DEM) data can be obtained from the Geospatial Data Cloud Platform (http://www.gscloud.cn; search term: “DEM Data”). Additional climate variables, including vapor pressure deficit and relative humidity, are available at https://doi.org/10.5281/zenodo.8070140. Note: Some datasets are hosted on Chinese‐language platforms. Non‐Chinese readers can use standard browser translation tools or contact the corresponding author for guidance.
