# Supplementary material for: Unraveling Spatiotemporal Heterogeneity and Driving Mechanism of Vegetation Recovery in Co‐Seismic Landslide Areas via Nonlinear Trajectories and Bayesian Networks
Source: Ecol Evol. 2026 Jul 29;16(8):e74117. doi: 10.1002/ece3.74117 (PMC13416765; doi:10.1002/ece3.74117)
Supplement: Supplementary file 1 — Table S1: Sensitivity analysis of recovery‐rate estimates under alternative trajectory‐phase definitions. Figure S1: Multicollinearity diagnostics for predictor variables. (a) Pearson correlation matrix for all initial variables. (b) VIF scores for the final selected variable set. Table S2: Sensitivity analysis of Bayesian Network predictive performance across different spatial block cross‐validation scales. Table S3: Derived prior and posterior probabilities of Fast Recovery under specific scenario interventions in the Bayesian Network. [file ECE3-16-e74117-s001.docx]

**Unraveling spatiotemporal heterogeneity and driving mechanism of vegetation recovery in co-seismic landslide areas via nonlinear trajectories and Bayesian networks**

Mingxuan Wan^1,2^, Wei Zhao^1, *^, Jiujiang Wu^1^, Yanqing Yang^1^, Junli Zhao^1,2^

^1^ Institute of Mountain Hazards and Environment, Chinese Academy of Sciences, Chengdu 610213, China.

^2^ University of Chinese Academy of Sciences, Beijing 100049, China.

*Corresponding author: Wei Zhao ([zhaow@imde.ac.cn)](mailto:email@address.edu))

Table S1. Sensitivity analysis of recovery-rate estimates under alternative trajectory-phase definitions.

| Buffer (Scale) | Mean Rate | SD |
| --- | --- | --- |
| 0.05 | 0.00825 | 0.02014 |
| 0.10 | 0.00825 | 0.02014 |
| 0.15 | 0.00905 | 0.01923 |
| 0.20 | 0.01146 | 0.01446 |
| 0.25 | 0.01218 | 0.01348 |

***Note:*** Mean fast-phase recovery rates (year⁻¹) and standard deviations are shown for different buffer scales defining the pixel-adaptive fast-regrowth phase. Buffer scale represents the proportion of the NDVI trajectory used as an edge buffer to avoid start/end effects. Values across 0.05–0.25 show that recovery-rate estimates are robust to reasonable variations in temporal-window definition. The 0.15 buffer scale was used as the reference configuration for consistency; it does not imply optimization.


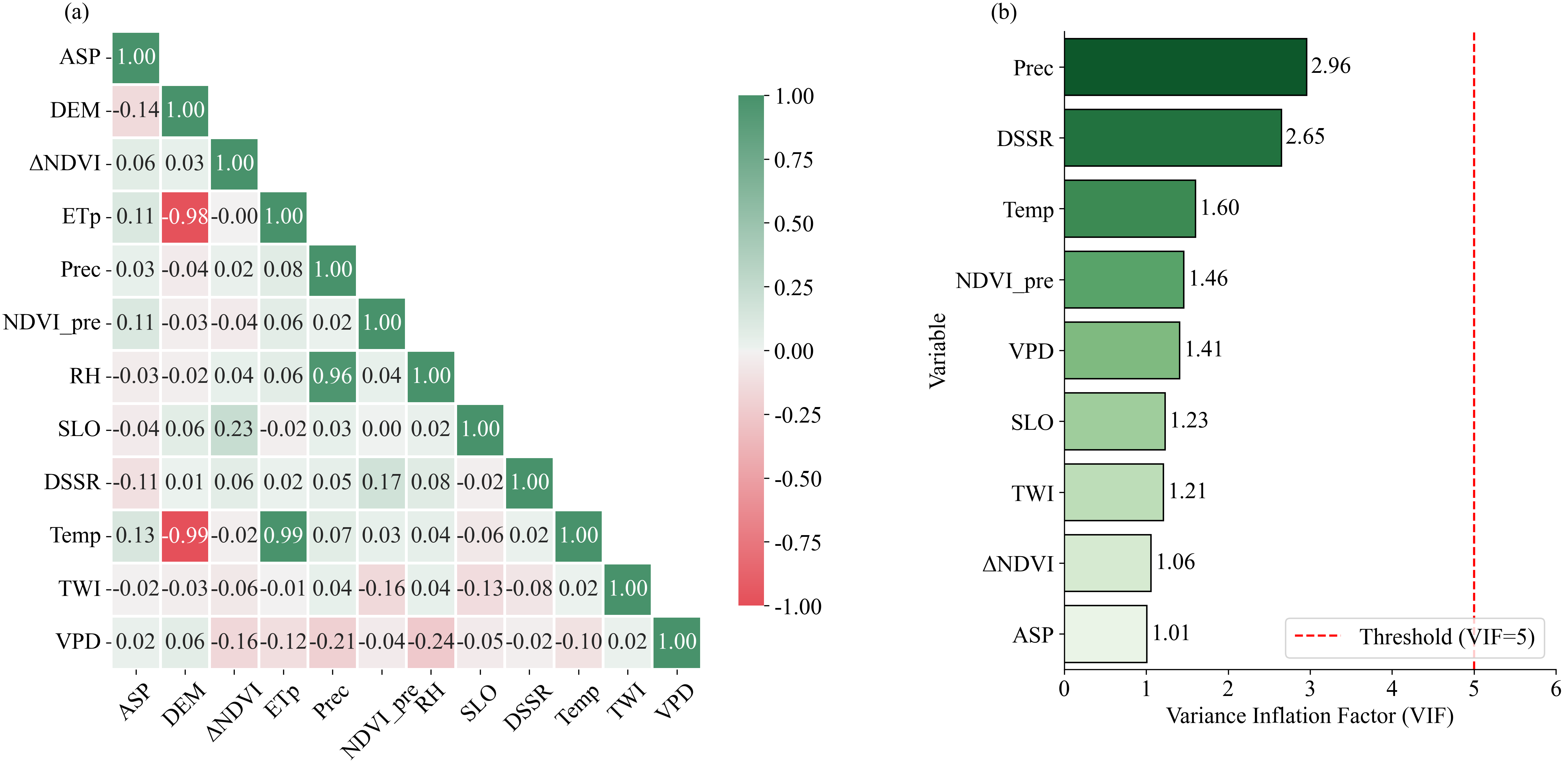


Fig S1. Multicollinearity diagnostics for predictor variables. (a) Pearson correlation matrix for all initial variables. (b) VIF scores for the final selected variable set.

Table S2. Sensitivity analysis of Bayesian Network predictive performance across different spatial block cross-validation scales.

| Spatial Block Scale | Error Rate (%) | Accuracy (%) | Quadratic Loss | Logarithmic Loss |
| --- | --- | --- | --- | --- |
| Baseline (Random) | 9.39 | 90.61 | 0.17 | 0.29 |
| 10 × 10 | 9.64 | 90.36 | 0.17 | 0.29 |
| 50 × 50 | 10.02 | 89.98 | 0.18 | 0.30 |
| 100 × 100 | 10.42 | 89.58 | 0.18 | 0.31 |
| 150 × 150 | 7.23 | 92.77 | 0.14 | 0.26 |
| 200 × 200 | 9.33 | 90.67 | 0.18 | 0.30 |

Table S3. Derived prior and posterior probabilities of Fast Recovery under specific scenario interventions in the Bayesian Network.

| Management Zone | Baseline Scenario  (NDVI_Pre / ΔNDVI) | Controllable Node | Baseline State | Prior  (P_pre,%) | Intervened State | Posterior  (P_post,%) | Δ P_recovery,% |
| --- | --- | --- | --- | --- | --- | --- | --- |
| Core Intervention | Medium/Low | Temp | Low | 6.58 | High | 19.30 | 12.72 |
| Core Intervention | Medium/High | Temp | Low | 27.80 | High | 43.90 | 16.10 |
| Core Intervention | Medium/Low | DSSR | Medium | 9.69 | Low | 26.10 | 16.41 |
| Core Intervention | Medium/High | DSSR | High | 32.00 | Low | 51.70 | 19.70 |
| Core Intervention | Medium/Low | Prec | Medium | 13.10 | High | 26.90 | 13.80 |
| Core Intervention | Medium/High | Prec | Medium | 35.00 | High | 45.50 | 10.50 |
| Potential Enhancement | Low/Low | Temp | Low | 15.90 | Medium | 39.00 | 23.10 |
| Potential Enhancement | Medium/Medium | Temp | Low | 12.30 | High | 34.10 | 21.80 |
| Potential Enhancement | Low/Low | DSSR | High | 10.60 | Low | 44.20 | 33.60 |
| Potential Enhancement | Medium/Medium | DSSR | High | 19.10 | Low | 36.90 | 17.80 |
| Potential Enhancement | Low/Low | Prec | High | 24.80 | Low | 34.30 | 9.50 |
| Potential Enhancement | Medium/Medium | Prec | Low | 23.70 | High | 40.50 | 16.80 |
